# The effects of postoperative radiotherapy on survival outcomes in patients under 65 with estrogen receptor positive tubular breast carcinoma

**DOI:** 10.1186/s13014-018-1177-9

**Published:** 2018-11-20

**Authors:** Jian-Xian Chen, Wen-Wen Zhang, Yong Dong, Jia-Yuan Sun, Zhen-Yu He, San-Gang Wu

**Affiliations:** 1 Department of Oncology, Division of Chemotherapy, the People’s Hospital of Baise, Baise, 533000 People’s Republic of China; 2 Department of Radiation Oncology, Sun Yat-sen University Cancer Center, State Key Laboratory of Oncology in South China, Collaborative Innovation Center of Cancer Medicine, Guangzhou, 510060 People’s Republic of China; 3 Department of Oncology, the 3rd People’s Hospital of Dongguan City, Dongguan, 523326 People’s Republic of China; 4 Department of Radiation Oncology, Xiamen Cancer Hospital, the First Affiliated Hospital of Xiamen University, Xiamen, 361003 People’s Republic of China

**Keywords:** Breast cancer, Estrogen receptors, Tubular carcinoma, Breast-conserving surgery, Adjuvant radiotherapy

## Abstract

**Background:**

The value of postoperative radiotherapy in tubular breast carcinoma patients under 65 years is uncertain.

**Methods:**

Data on patients with estrogen receptor positive T1N0M0 tubular breast carcinoma who were younger than 65 years and who received breast-conserving surgery between 2000 and 2013 were retrieved from the Surveillance, Epidemiology and End Results database. Demographic, clinicopathologic features, and receipt of postoperative radiotherapy were analyzed to investigate effects on survival.

**Results:**

Data from 2442 patients were analyzed, of whom 2020 (82.7%) received postoperative radiotherapy and 422 (17.3%) did not. The number of patients treated with or without postoperative radiotherapy showed no differences during the study period (*p* = 0.184). Radiotherapy was more likely to be administered in patients with well differentiated tumors. Multivariate Cox analysis showed that postoperative radiotherapy delivery was significantly correlated with better breast cancer-specific survival (BCSS) (hazard ratio [HR] 0.297, 95% confidence interval [CI] 0.105–0.836, *p* = 0.022) and overall survival (OS) (HR 0.656, 95% CI 0.441–0.978, *p* = 0.038). Ten 10-year BCSS was 99.3% in patients who received postoperative radiotherapy and 98.1% in those who did not (*p* = 0.020), and 10-year OS was 93.4 and 91.0%, respectively (*p* = 0.029). Postoperative radiotherapy increased BCSS and OS in the subgroups of age < 50 years, non-Hispanic white, well differentiated tumors, and progesterone receptor positive tumors.

**Conclusions:**

Postoperative radiotherapy after breast-conserving surgery improved survival outcomes in tubular breast carcinoma patients aged < 50 years. However, omitting postoperative radiotherapy may not decrease survival in patients aged ≥50 years.

## Background

Postoperative adjuvant radiotherapy has been confirmed to improve local recurrence rates and reduce breast cancer-related death in patients with node-negative and node-positive breast cancer after breast-conserving surgery [1]. However, these results reflect the outcomes of patients with invasive breast cancer without further analysis of the impact of histological subtypes on outcomes [1]. Tubular breast carcinoma is a rare, well-differentiated invasive breast carcinoma; whether a lesion is classified as pure or mixed tubular breast carcinoma depends on its tubular composition, nuclear grade, and mitotic activity [2–5]. The clinicopathological characteristics of tubular breast carcinoma include small tumor size, node negativity, low tumor grade, and hormone receptor positive disease [6]. The 10-year locoregional recurrence rate, breast cancer-specific survival (BCSS), and overall survival (OS) in this disease have been reported as 4.7, 97.2–100%, and 90–97%, respectively [7–16].

Breast-conserving surgery is the main local treatment for tubular breast carcinoma. A previous study showed that postoperative radiotherapy was not associated with better BCSS when compared with surgery alone in patients older than 65 years [14], and this was supported by results in other invasive breast cancers [17]. However, given the excellent survival rates of patients with tubular breast carcinoma, it is still controversial whether patients younger than 65 years should be treated with postoperative radiotherapy as with other invasive breast cancers [8, 16, 18–21]. The aim of the present study was to assess the impact of postoperative radiotherapy on survival outcomes in tubular breast carcinoma patients aged less than 65 years after breast-conserving surgery using a population-based cancer database.

## Patients and methods

### Patients

Data on patients who were diagnosed with tubular breast carcinoma from 2000 to 2013 were retrieved from the Surveillance, Epidemiology and End Results (SEER) database [22]. Inclusion criteria were: (1) female patients with pathologically confirmed tubular breast carcinoma, (2) aged less than 65 years, (3) tumor size ≤2 cm (T1 classification) and node-negative disease, (4) estrogen receptor (ER) positive cancer, (5) breast-conserving surgery, (6) complete information on race/ethnicity, tumor grade, progesterone receptor (PR) status, chemotherapy (no/unknown, or yes), and postoperative radiotherapy delivery (no/unknown, or beam radiotherapy). As the SEER database consists of de-identified information, the study was exempt from the approval process of Institutional Review Boards of the First Affiliated Hospital of Xiamen University.

### Statistical analysis

The Pearson’s chi squared test was used to compare distributions of variables between the groups. Binary logistic regression was used to evaluate predictors for receiving postoperative radiotherapy. Ten-year BCSS and OS curves and rates were estimated and then compared using the Kaplan–Meier method, followed by log-rank tests. A multivariate Cox proportional hazards model including all variables was used to calculate adjusted hazard ratios (HRs) and their corresponding 95% confidence intervals (CIs) in the entire cohort, and the Backward Wald method was used in each risk group. SPSS version 22 (IBM Corporation, Armonk, NY, USA) was used to perform all statistical tests, and a *p* value of < 0.05 was considered to be statistically significant.

## Results

A total of 2442 patients met the inclusion criteria, of whom 2020 (82.7%) had received postoperative radiotherapy and 422 (17.3%) had not. The median age was 54 years (range 28–64 years). Most of patients were non-Hispanic white, and the majority of tumors were well differentiated, ≤1 cm, and PR positive. Only 5.7% of patients had received chemotherapy (Table 1). The number of patients with or without postoperative radiotherapy delivery showed no differences during the study period (*P* = 0.184).

**Table 1:** Patient characteristics

Variables	*n*	no-RT (%)	RT (%)	*p*
Age (years)				
< 50	682	112 (26.5)	570 (28.2)	0.512
≥ 50	1760	310 (73.5)	1450 (71.8)	
Race/ethnicity				
Non-Hispanic white	2098	357 (84.6)	1741 (86.2)	0.463
Non-Hispanic black	103	21 (5.0)	82 (4.1)	
Hispanic	133	28 (6.6)	105 (5.2)	
Other	108	16 (3.8)	92 (4.6)	
Grade				
Well differentiated	2244	380 (90.0)	1864 (92.2)	0.012
Moderately differentiated	176	33 (7.8)	143 (7.1)	
Poorly/undifferentiated	22	9 (2.1)	13 (0.6)	
Tumor size				
T1mic	22	6 (1.4)	16 (0.8)	0.444
T1a	662	122 (28.9)	540 (26.7)	
T1b	1154	196 (46.4)	958 (47.4)	
T1c	604	98 (23.2)	506 (25.0)	
PR status				
Negative	328	47 (11.1)	281 (13.9)	0.136
Positive	2114	375 (88.9)	1739 (86.1)	
Chemotherapy				
No/unknown	2302	391 (92.7)	1911 (94.6)	0.134
Yes	140	31 (7.3)	109 (5.4)	

*PR* progesterone receptor, *RT* radiotherapy, *T* tumor

Multivariable binary logistic regression was used to find indicators that were independently associated with postoperative radiotherapy delivery (Table 2). The results showed an increasing use of postoperative radiotherapy in patients with well differentiated tumors. Age, race/ethnicity, tumor size, PR status, and chemotherapy were not associated with the administration of postoperative radiotherapy.

**Table 2:** Multivariable logistic regression analysis for factors that predict receiving postoperative radiotherapy

Variables	OR	95% CI	*p*
Age (years)			
< 50	1		
≥ 50	0.874	0.685–1.114	0.276
Race/ethnicity			
Non-Hispanic white	1		
Non-Hispanic black	0.785	0.479–1.287	0.337
Hispanic	0.746	0.483–1.153	0.187
Other	1.206	0.699–2.083	0.501
Grade			
Well differentiated	1		
Moderately differentiated	0.883	0.596–1.310	0.583
Poorly/undifferentiated	0.294	0.125–0.694	0.005
Tumor size			
T1c	1		
T1mic	0.560	0.209–1.500	0.249
T1a	0.802	0.595–1.081	0.148
T1b	0.901	0.687–1.182	0.451
PR status			
Negative	1		
Positive	0.750	0.538–1.047	0.091
Chemotherapy			
No/unknown	1		
Yes	0.688	0.448–1.057	0.088

*CI* confidence interval*, OR* odds ratio, *PR* progesterone receptor, *T* tumor

Multivariate Cox analysis was conducted after adjusting for age, race/ethnicity, disease grade, tumor size, PR status, chemotherapy, and postoperative radiotherapy. Postoperative radiotherapy delivery was found to be significantly related to better BCSS (HR 0.297, 95% CI 0.105–0.836, *p* = 0.022) and OS (HR 0.656, 95% CI 0.441–0.978, *p* = 0.038). In addition, PR-positive tumor status was correlated with better BCSS (HR 0.330, 95% CI 0.112–0.968, *p* = 0.043), while older age (≥50 years) was associated with poorer OS (HR 2.030, 95% CI 1.275–3.231, *p* = 0.003) (Table 3).

**Table 3:** Multivariate Cox regression analysis of survival outcomes

Variables	BCSS			OS		
	HR	95% CI	*p*	HR	95% CI	*p*
Age (years)						
< 50	1			1		
≥ 50	0.708	0.227–2.008	0.552	2.03	1.275–3.231	0.003
Race/ethnicity						
Non-Hispanic white	1			1		
Non-Hispanic black	1.825	0.225–14.794	0.573	1.162	0.473–2.855	0.744
Hispanic	–	–	0.992	0.986	0.433–2.245	0.974
Other	2.167	0.277–16.936	2.167	0.600	0.190–1.892	0.383
Grade						
Well differentiated	1			1		
Moderately differentiated	–	–	0.986	1.107	0.607–2.019	0.740
Poorly/undifferentiated	–	–	0.996	1.954	0.614–6.220	0.257
Tumor size						
T1c	1			1		
T1mic	–	–	0.998	–	–	0.956
T1a	0.488	0.087–2.736	0.415	0.648	0.385–1.089	0.101
T1b	1.066	0.319–3.560	0.918	0.943	0.629–1.415	0.778
PR status						
Negative	1			1		
Positive	0.330	0.112–0.968	0.043	0.712	0.464–1.092	0.120
Chemotherapy						
No/unknown	1			1		
Yes	2.454	0.489–12.314	0.275	1.781	0.951–3.336	0.071
Radiotherapy						
No	1			1		
Yes	0.297	0.105–0.836	0.022	0.656	0.441–0.978	0.038

*BCSS* breast cancer-specific survival, *HR* hazard ratio, *CI* confidence interval, *OS* overall survival, *PR* progesterone receptor

**Fig. 1:**
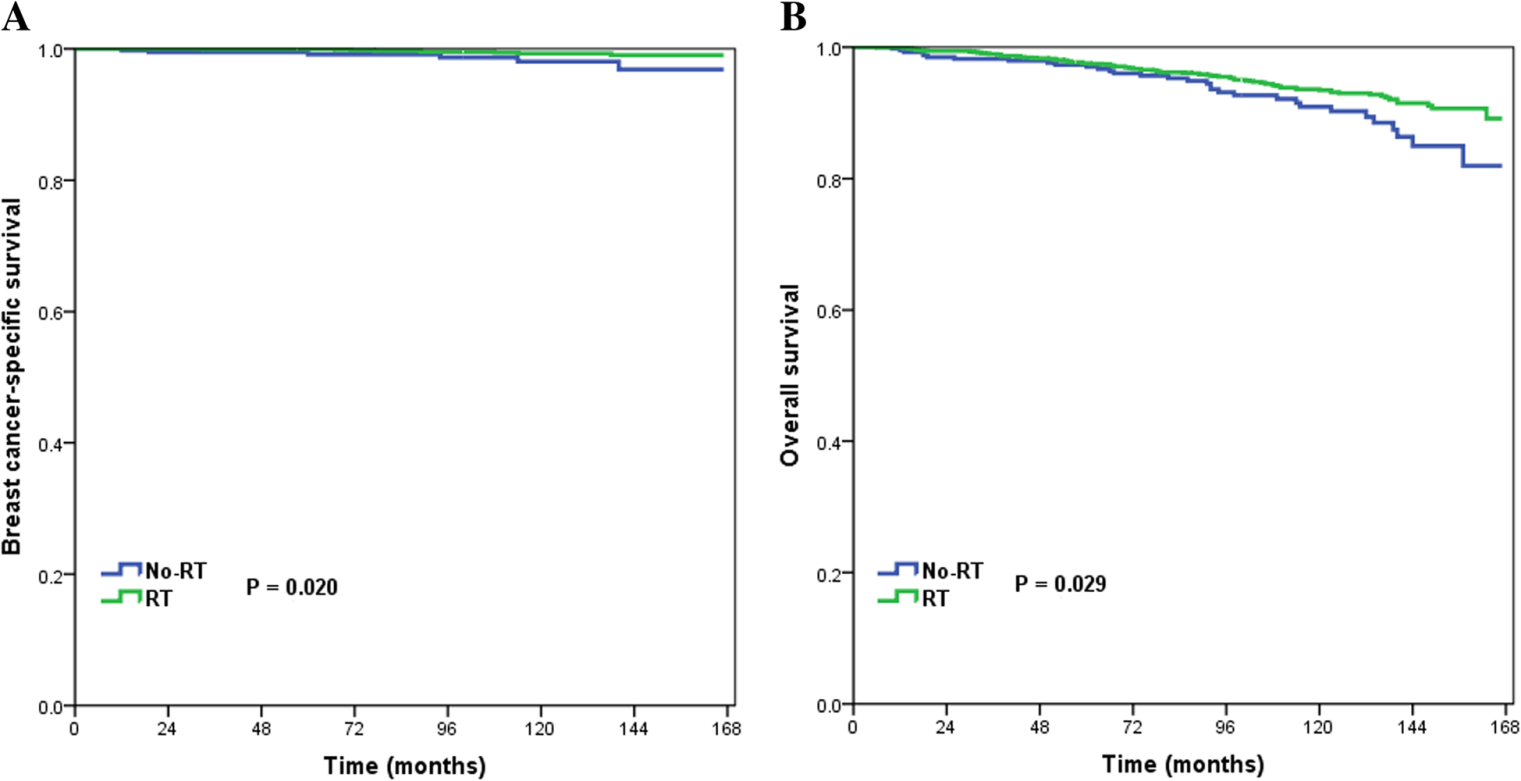
Breast cancer-specific survival and overall survival according to whether or not postoperative radiotherapy was received after breast conserving surgery

**Fig. 2:**
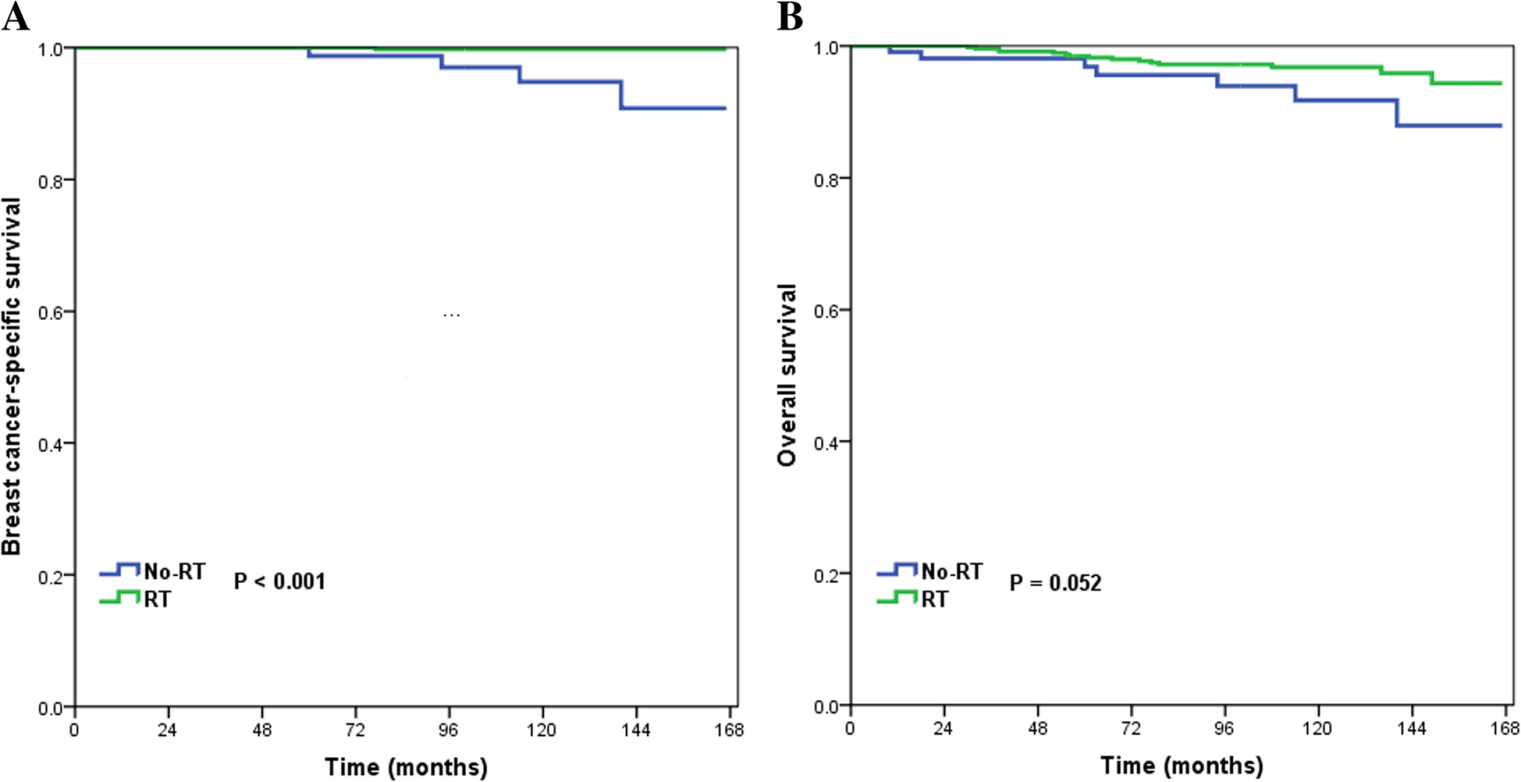
Breast cancer-specific survival and overall survival in patients aged < 50 years according to whether or not postoperative radiotherapy was received after breast conserving surgery

Ten-year BCSS and OS were compared between the no-radiotherapy and radiotherapy groups for all patients and for each variable. For all patients, 10-year BCSS was 99.3% in patients who had postoperative radiotherapy and 98.1% in those who did not (log-rank test, *p* = 0.020) (Fig. 1a), and 10-year OS values were 93.4 and 91.0%, respectively (log-rank test, *p* = 0.029) (Fig. 1b). In non-Hispanic white patients, those with well differentiated tumors, and those with PR-positive tumors, postoperative radiotherapy was significantly related to better BCSS and OS (Table 4). Postoperative radiotherapy delivery was also significantly associated with better BCSS in patients aged < 50 years (log-rank test, *p* = 0.001), and there was a borderline trend of improving OS (log-rank test, *p* = 0.052) in these patients. Multivariate Cox analysis in each variable group showed that postoperative radiotherapy increased BCSS and OS in patients aged < 50 years (Fig. 2a and Fig. 2b), non-Hispanic white patients, and those with well differentiated and PR-positive tumors (Table 5).

**Table 4:** 10-year breast cancer-specific survival and overall survival by variable

Variables	BCSS			OS		
	no-RT (%)	RT (%)	*p*	no-RT (%)	RT (%)	*p*
Age (years)						
< 50	94.8	99.7	0.001	91.8	96.8	0.052
≥ 50	99.3	99.1	0.836	90.7	92.2	0.135
Race/ethnicity						
Non-Hispanic white	97.8	99.4	0.005	90.2	93.4	0.011
Non-Hispanic black	100	97.7	0.616	93.8	90.9	0.949
Hispanic	100	100	1	96.0	92.7	0.777
Other	100	98.7	0.691	100	96.2	0.479
Grade						
Well differentiated	97.8	99.2	0.017	91.2	93.6	0.038
Moderately, poorly/undifferentiated	100	100	1	89.1	92.0	0.506
Tumor size						
≤ 1.0 cm	98.7	99.3	0.101	92.6	93.8	0.121
> 1.0 cm and ≤ 2.0 cm	96.1	99.2	0.07	86.2	92.3	0.114
PR status						
Negative	100	97.4	0.705	87.2	89.0	0.434
Positive	97.8	99.6	0.008	91.4	94.3	0.029

*BCSS* breast cancer-specific survival, *OS* overall survival, *PR* progesterone receptor, *RT* radiotherapy

**Table 5:** Multivariate Cox regression analysis of the effect of postoperative radiotherapy on breast cancer-specific survival and overall survival by variable

Variables	BCSS			OS		
	Adjusted HR of RT	95% CI	*p*	Adjusted HR of RT	95% CI	*p*
Age (years)						
< 50	0.042	0.005–0.393	0.005	0.396	0.161–0.971	0.043
≥ 50	0.746	0.155–3.588	0.715	0.726	0.463–1.139	0.163
Race/ethnicity						
Non-Hispanic white	0.239	0.080–0.711	0.01	0.612	0.404–0.927	0.020
Non-Hispanic black	0.375		0.997	0.558	0.049–6.369	0.638
Hispanic	–	–	–	2.108	0.225–19.747	0.514
Other	1.564	–	0.981	–	–	0.976
Grade						
Well differentiated	0.287	0.102–0.809	0.018	0.647	0.423–0.990	0.045
Moderately, poorly/undifferentiated	–	–	–	0.615	0.180–2.096	0.439
Tumor size						
≤ 1.0 cm	0.340	0.098–1.175	0.088	0.698	0.432–1.129	0.143
> 1.0 cm and ≤ 2.0 cm	0.172	0.024–1.239	0.081	0.543	0.259–1.140	0.107
PR status						
Negative	0.648	0.056–7.445	0.727	0.646	0.223–1.791	0.404
Positive	0.204	0.059–0.705	0.012	0.631	0.407–0.978	0.039

*BCSS* breast cancer-specific survival, *HR* hazard ratio, *CI* confidence interval, *OS* overall survival, *PR* progesterone receptor, *RT* radiotherapy

## Discussion

The present study investigated the role of postoperative radiotherapy in ER-positive T1 N0 tubular breast carcinoma in patients younger than 65 years. The results indicated that postoperative radiotherapy was associated with better outcomes in these patients, especially in the subgroups of patients aged < 50 years, non-Hispanic white patients, and those with well differentiated and PR-positive tumors.

Results from the Early Breast Cancer Trialists’ Collaborative Group showed absolute benefits of 15.4% in local control and 3.3% in BCSS in patients who received postoperative radiotherapy after breast-conserving surgery [1]. Postoperative radiotherapy is therefore the standard treatment for breast cancer after breast-conserving surgery. Our previous study including tubular breast carcinoma patients with aged ≥65 years found that the rate of the postoperative radiotherapy administration steadily declined from 2000 to 2013 [14]. A previous SEER study indicated that 70.4% of all-age patients who received breast-conserving surgery were treated with postoperative radiotherapy between 1992 and 2007 [20]. In our study, 82.7% of aged < 65 years patients were received postoperative radiotherapy, and the postoperative radiotherapy administration rate was no significantly difference during the study period. The difference in the postoperative radiotherapy administration rate by different age groups may be due to the lack of survival benefit in elderly invasive breast carcinoma patients without postoperative radiotherapy, while an increased mortality of younger invasive breast carcinoma patients who omitted of radiotherapy after breast-conserving surgery [23–25]. In addition, several studies in recent years have shown a wide range of the rate of postoperative radiotherapy receipt (43–93%) in tubular breast carcinoma patients [7, 10, 16, 18, 26]. Therefore, there is still controversy over whether to use postoperative radiotherapy for tubular breast carcinoma.

Since most of the patients with tubular breast carcinoma in the previous studies had T1 tumors, negative lymph nodes, and were ER positive, and because these studies only included small numbers of patients, identifying subgroups that were more likely to receive postoperative radiotherapy was difficult. In our study, patients who received postoperative radiotherapy had a lower probability of having poor or undifferentiated tumors; a similar result to a previous SEER study [20]. It is difficult to explain why patients with poor or undifferentiated tumors were less likely to receive postoperative radiotherapy. Our study only included 2.1 and 0.6% of patients with poor or undifferentiated tumors in the no-radiotherapy and radiotherapy groups, respectively. We believe that this may be related to the disease characteristics of tubular breast carcinoma, because over 90% of patients had well differentiated tumors.

In clinical practice, the rate of locoregional recurrence was the determining factor for making a decision about postoperative radiotherapy. The SEER program does not record patterns of locoregional recurrence; however, a study of tubular breast carcinoma including 11 retrospective series between 1979 and 2005 showed a significant improvement in locoregional recurrence rates with postoperative radiotherapy compared with surgery alone (3.4% vs. 8.3%, *p* = 0.005) [21]. In another literature review including recent series between 2001 and 2012, the results indicated that the mean locoregional recurrence rate in the radiotherapy group was 4.1% (range, 0–7%), and 8.4% (range, 0–28%) in the no-radiotherapy group [16]. Locoregional recurrence may not affect BCSS [7]. However, there was heterogeneity in the study cohorts with respect to age, central pathology review, and histological inclusion criteria. In addition, some new tumors may be phenotypically different from the primary tumors, while other new tumors may be new primary tumors [7, 27]; it is therefore difficult to distinguish whether the breast tumor is a true recurrence or a new primary lesion.

In a previous study in a South Korean population including 70 patients with tubular breast carcinoma, only 1 patient developed locoregional recurrence with invasive ductal carcinoma after breast-conserving surgery [10]. In another study of 205 tubular breast carcinoma patients, 7 patients developed disease recurrence during the follow-up period, including 1 patient with a tumor bed recurrence of invasive ductal carcinoma, 3 patients with bone metastases, and 5 patients with contralateral breast cancer (1 patient with tubular breast carcinoma and 4 patients with invasive ductal carcinoma) [18]. Therefore, recurrence of tubular breast carcinoma may often not be true recurrence of tubular breast carcinoma, instead being the result of the long survival time of patients who go on to develop secondary breast cancer.

In patients younger than 65 years, postoperative radiotherapy remains the standard treatment after breast-conserving surgery. However, this recommendation is applies to invasive breast cancer in general. As tubular breast carcinoma has an excellent prognosis, the role of postoperative radiotherapy is still controversial. In a large cohort from Florence (307 patients, median age 56 years), postoperative radiotherapy after breast-conserving surgery did not lead to a survival benefit [8]. Another study in a South Korean population (median age 48 years) found that administration of postoperative radiotherapy was not correlated with better recurrence-free survival in a multivariate analysis [18]. However, in a study by Hansen et al. which included 115 tubular breast carcinoma patients who received breast-conserving surgery (median age 55 years), the 5-year ipsilateral breast recurrence-free survival rate was 100% in patients who had postoperative radiotherapy and 89% in those who did not (*p* < 0.001) [16]. A study by Fritz et al. confirmed that postoperative radiotherapy was associated with survival benefit in tubular breast carcinoma patients after breast-conserving surgery: the 10-year survival rate in the radiotherapy group was 85.9% compared with 76.3% in the no-radiotherapy group (median age 58 years, *p* = 0.035) [19]. In addition, findings by Sullivan et al. suggested that postoperative radiotherapy in younger tubular breast carcinoma patients was beneficial for local control [21]. However, heterogeneity of tubular breast carcinoma data may not allow for accurate comparisons between these studies.

In the present study, only patients under 65 years old were included. Patients who received postoperative radiotherapy had better 10-year BCSS and OS, but the absolute survival benefits were only 1.2 and 2.4%, respectively. This is similar to a previous study from SEER [20], which also found a 3% absolute OS benefit for postoperative radiotherapy 10 years after breast-conserving surgery (there was no further analysis of the impact on BCSS). The current study found that the BCSS benefit was similar to that found for invasive breast cancer in the study from the Early Breast Cancer Trialists’ Collaborative Group, in which the 15-year BCSS benefit was 3.3% [1]. Our results showed that postoperative radiotherapy improved survival in tubular breast carcinoma patients younger than 65 years, but that the absolute survival benefit was small. Therefore, the pros and cons of radiotherapy in these patients should be considered when making the decision about whether to carry out this treatment. In addition, studies including gene expression profiling, such as 21-gene recurrence score test could potentially identify a subgroup of patients who may benefit from addition or omission of postoperative radiotherapy [28, 29].

Multivariate analysis in the present study found that older patients (≥50 years) was negatively correlated with OS but that age had no effect on BCSS. In addition, race/ethnicity, tumor grade, tumor size, PR status, and chemotherapy had no significant effect on survival outcomes. Patients aged < 50 years, non-Hispanic white patients, and those with well differentiated and PR-positive tumors had improved survival rates with postoperative radiotherapy. Since most of the patients in the study were non-Hispanic white, and had well differentiated, PR-positive tumors, we believe that the survival benefits for these subgroups may be related to the demographic and clinicopathological characteristics of the disease itself. In our study, 72.1% of patients were aged ≥50 years, but we found that postoperative radiotherapy delivery did not improve survival in this subgroup. Our previous study also found that postoperative radiotherapy did not produce a survival benefit in patients aged ≥65 years [14]. Therefore, it may be safe to avoid postoperative radiotherapy in women aged ≥50 years after breast-conserving surgery for tubular breast carcinoma.

There are several limitations to this study. First, there is inherent bias in any retrospective study. Propensity score-matching can be used to decrease the potential effect of selection bias. However, the only significant difference in patient characteristics found between the radiotherapy and no-radiotherapy groups was in tumor grade, and so propensity score-matching was not used. Second, two subtypes of tubular breast carcinoma have been described: pure and mixed. However, previous studies have shown that outcomes are similar in these two subtypes [30, 31]. Third, the SEER database lacked information on centralized pathologic review, details of radiotherapy, chemotherapy, hormonal therapy, and treatment outcomes including locoregional control and distant metastases. In addition, it has been shown that there are many inaccuracies in the SEER database, with high rates of under-reporting for radiotherapy administration [32, 33].

## Conclusion

In conclusion, our results suggest that in patients with tubular breast carcinoma aged < 65 years, postoperative radiotherapy improves survival outcomes in patients aged < 50 years. However, omitting postoperative radiotherapy may not decrease survival in patients aged ≥50 years. More prospective studies are needed to confirm these findings.

## Abbreviations

BCSS: Breast cancer-specific survival
CIs: Confidence intervals
ER: Estrogen receptor
HRs: Hazard ratios
OS: Overall survival
PR: Progesterone receptor
SEER: Surveillance, Epidemiology and End Results
